# Avian wings can lengthen rather than shorten in response to increased migratory predation danger

**DOI:** 10.1002/ece3.10325

**Published:** 2023-07-22

**Authors:** Ronald C. Ydenberg, Guillermo Fernández, Enver Ortiz Lopez, David B. Lank

**Affiliations:** ^1^ Centre for Wildlife Ecology, Department of Biological Sciences Simon Fraser University Burnaby British Columbia Canada; ^2^ Unidad Académica Mazatlán, Instituto de Ciencias del Mary Limnología Universidad Nacional Autónoma de México Mazatlan Mexico; ^3^ Centro de Ornitología y Biodiversidad (CORBIDI) Lima Peru

**Keywords:** migration, morphology, peregrine falcon, predation danger, sandpipers, wing shape

## Abstract

Increasing predation danger can select for safety‐enhancing modifications to prey morphology. Here, we document the multi‐decade wing lengthening of a Pacific flyway migrant, the western sandpiper (*Calidris mauri*), and contrast this with contemporaneous wing shortening of the closely related semipalmated sandpiper (*C. pusilla*) on the Atlantic flyway. We measured >12,000 southbound western sandpipers captured from 1978 to 2020 at a major stopover site in British Columbia. Wing length increased at 0.074 mm year^−1^ (SE = 0.017; *p* < .0003) for adults, and 0.087 mm year^−1^ (SE = 0.029; *p* < .007) for juveniles. These rates are of similarly large magnitude (4%–5% overall), but opposite in direction, to the rate we previously reported for semipalmated sandpiper adults (−0.103 mm year^−1^). In both species, the change is specific to wings rather than being part of a general body size change. We interpret both trends as responses to the ongoing strong increase of peregrine falcon (*Falco peregrinus*) populations since the mid‐1970s, an important predator encountered by these species in contrasting ways during migration. Western sandpipers and peregrine migrations have temporal and spatial overlap. Longer wings enhance migratory speed and efficiency, enabling western sandpipers to decrease overlap by advancing to safer zones ahead of falcon passage. In contrast, semipalmated sandpipers primarily encounter peregrines as residents at migratory staging sites. Shorter wings improve acceleration and agility, helping migrants to escape attacks. Juvenile western sandpiper wing length also shows a component additive to the lengthening trend, shifting between years at 0.055 mm day^−1^ with the highly variable snowmelt date, with wings shorter following early springs. On the Pacific flyway, the timing of peregrine southward passage advances with snowmelt, increasing the relative exposure of juveniles to post‐migratory resident peregrines. We interpret this annual wing length adjustment as an induced defense, made possible because snowmelt timing is a reliable cue to danger in the upcoming migration.

## Graphical abstract

Avian wing lengths can lengthen or shorten in response to increased predation danger depending on whether the danger is primarily from migratory or resident predators encountered during migration. Danger from migratory predators favors longer wings to increase the speed of migration while danger from resident predators favors shorter wings to increase escape probability.

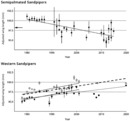

## 1 INTRODUCTION

Experiments in micro‐ and mesocosms demonstrate that an increase in the presence of predators can lead to safety‐enhancing changes in the behavior and morphology of prey (Brönmark & Miner, 1992; Hoverman et al., 2005; McCollum & Leimberger, 1997; Oufiero et al., 2011). These experiments demonstrate both rapid microevolutionary and phenotypically plastic responses (O'Steen et al., 2002; Reznick et al., 1990). Field studies (DeWitt et al., 2019; Hammerschlag et al., 2018) have documented findings consistent with these results, though experimental verification at a large scale in nature is more difficult to acquire (Sheriff et al., 2020). Here we consider the hypothesis that ongoing wing length changes of two migratory sandpiper species are safety‐enhancing responses to raptor population recovery.

Many papers describe animal morphological change over recent decades, including at least 12 papers documenting long‐term directional changes in the wing length of bird species (Table 1). Climate change is invoked as the causal factor in some of these studies (1–5 in Table 1). Others (6–10 in Table 1) invoke changing environmental demands on flight performance attributes. Flight speed, power requirement, acceleration, climb rate, agility, and energy efficiency are all strongly influenced by wing morphology (Pennycuick, 1989). A change in the relative importance of these performance characteristics in any type of flight (foraging, display, migration, predator escape; Marchetti et al., 1995) would exert corresponding selection pressure on wing morphology. Examples include large‐scale habitat change requiring more or less flight (Desrochers, 2010), altered migration routing or timing requiring more extended or faster travel (Hahn et al., 2016), and changed predator abundance that alters the importance of escape ability.

**TABLE 1:** Studies reporting a change in avian wing morphology over recent decades.

Species	Location	Years	Result	Attributed to	Source
1. Passerines 7 species	England – two sites	1968–2003	Mixed WL and BM changes, differs between sites	Climate change	Yom‐Tov et al. (2006)
2. Passerines 101 species	Pennsylvania	1961–2006	WL and BM show small decline across all species	Climate change	van Buskirk et al. (2010)
3. Passerines 41 migrant species	California	1971–2010	WL increase; some BM increases	Higher climate variability	Goodman et al. (2012)
4. Passerines 24 species	Australia	1960–2007	Nine species decrease WL; five species increase WL; others reverse direction	Climate change	Gardner et al. (2014)
5. Passerines 52 migrant species	Chicago	1978–2016	WL increase; tarsus length decrease	Climate change	Weeks et al. (2019)
6. Passerines 21 species	e. North America	1900–2008	11/21 change wing shape; more pointed in the boreal region—deforestation so more flight less pointed in the temperate region—afforestation so less flight	Habitat isolation	Desrochers (2010)
7. Cliff swallow (breeding)	Nebraska	1984–2012	WL decrease; more traffic, selection for higher maneuverability		Brown and Brown (2013)
8. Semipalmated Sandpiper	Nearctic	1970–2015	WL increase prior to 1980; subsequent steady decrease, no bill length change	Peregrine recovery selection for better escape performance	Lank et al. (2017)
9. Calidridines 3 migrant species	James Bay Ontario	1980–2015	WL decrease; wings more convex; no bill length change	Environmental change selecting for higher maneuverability	Anderson et al. (2019)
10. Passerines 31 neotropical migrants	Maryland	1980–2012	Variable body size change, WL and BM increase	None	Collins et al. (2017)
11. 11 species	Germany	1889–2010	Variable body size change	No support for climate change	Salewski, Siebenrock, et al. (2014)
12. Stonechat *Saxicola torquata*	Germany	1989–2012	WL increase, tail length decline	Climate change conclusion premature	Salewski, Hochachka, and Flinks (2014)

Abbreviations: BM, body mass; WL, wing length.

This study focuses on wing morphology with respect to migration and predation danger. Wing shape embodies a trade‐off between traits that improve long‐distance flight performance against those that improve predator escape ability (Hahn et al., 2016; Phillips et al., 2018). Relatively long, pointed, and concave wings have less drag and are more energy efficient, thus supporting faster overall migratory progression (distance per day). In contrast, relatively short, round, and convex wings provide more lift, acceleration, and agility, thus improving escape performance during predator attacks (Burns, 2003; Hedenström & Møller, 1992; Pennycuick et al., 1994; Swaddle & Lockwood, 1998; Vágási et al., 2016).

The selective balance of defensive attributes can shift with predator population size. Gosler et al. (1995) investigated multi‐decade changes in the mid‐winter body mass of the great tit (*Parus major*) in England in relation to changes in predator abundance, based on the idea that greater body mass (due to extra fat) reduces starvation but raises predation risk. Mid‐winter body mass rose as sparrowhawk (*Accipiter nisus*) populations were decimated by intense DDT usage beginning in the late 1940s, and then fell in the 1970s as sparrowhawk populations recovered.

Raptor numbers in North America followed a similar trajectory. Peregrines (*Falco peregrinus*) were threatened with extirpation over large parts of their range until the mid‐1970s, when the decline reversed following DDT regulation, the enactment of endangered species legislation, and the initiation of many conservation measures, including introductions of captive‐bred birds into the wild. These steps proved effective and the peregrine was removed from the U.S. endangered species list in 1999 (Cade & Burnham, 2003). Other raptor species have also increased since the mid‐1970s, and the ongoing upward trend of raptor numbers in North America contrasts strongly with the declines of most other groups of birds (Rosenberg et al., 2019).

Lank et al. (2017) considered the effect of steadily climbing peregrine numbers on the wing size and shape of migratory semipalmated sandpipers (*Calidris pusilla*), assuming that shorter, rounder wings to facilitate escape would be increasingly favored. They show that adult wings shortened by ~3.8 mm from 1980 to 2015. The change cannot be accounted for by greater declines of larger‐bodied eastern populations of this species (Bliss et al., 2019), as had originally been conjectured (Hicklin & Chardine, 2012). An independent study documents similar wing shortening in two other long‐distance Atlantic flyway migrant sandpiper species, as well as for juvenile semipalmated sandpipers (Anderson et al., 2019). Brown and Brown (2013) earlier proposed a similar hypothesis to explain wing shortening in the cliff swallow (*Petrochelidon pyrrhonota*), conjecturing that increased danger from road traffic selected for shorter wings because they provide greater agility and higher acceleration. Salewski, Hochachka, and Flinks (2014) list “increasing predation pressure” as a hypothesis potentially accounting for changing wing morphology of stonechats (*Saxicola torquata*) and note that this idea has yet to be tested.

**FIGURE 1:**
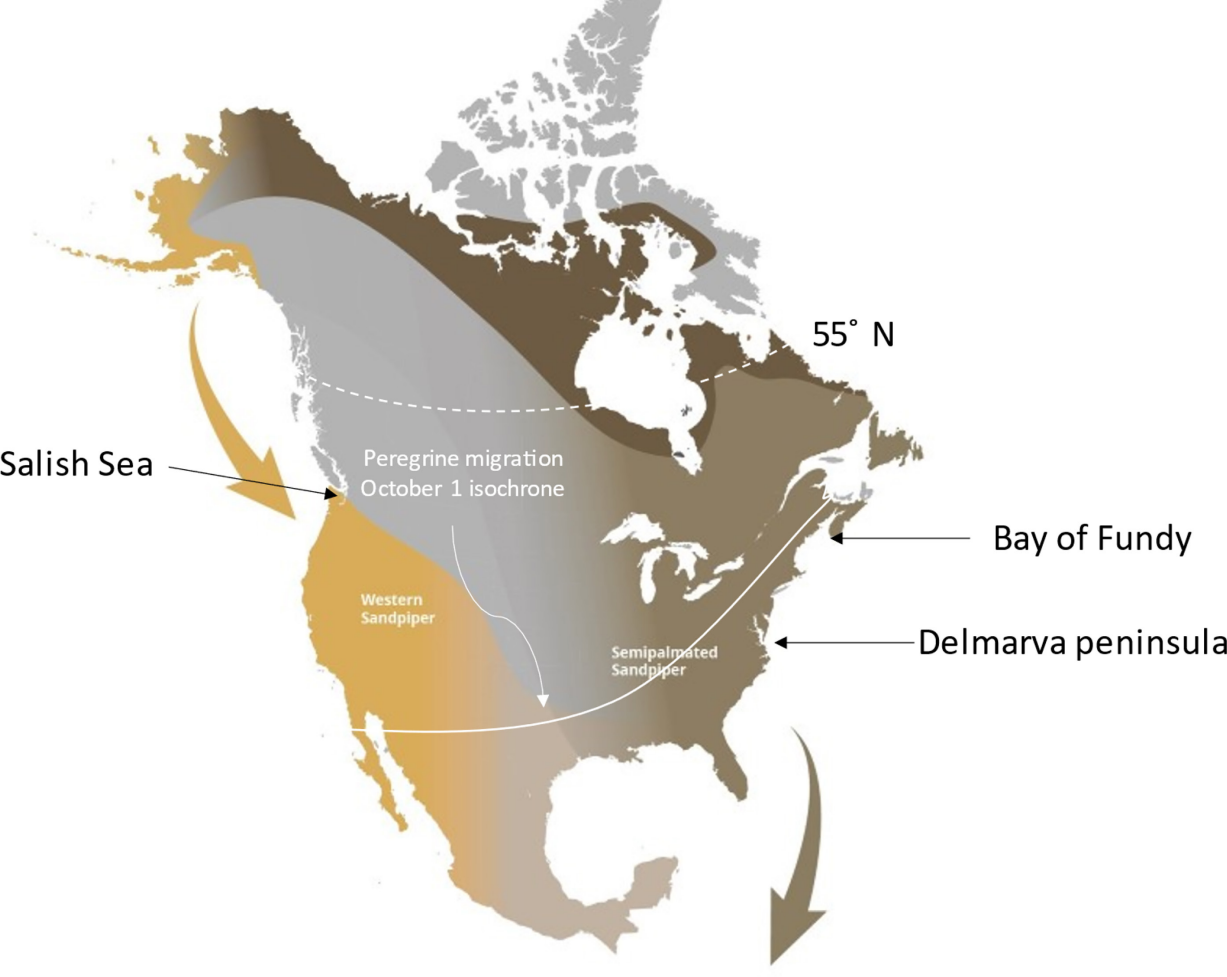
Geography of the migratory interaction of sandpipers and falcons in North America. Summary phenological data in Table 2 were collected in the Salish Sea and the Bay of Fundy. Peregrines breed across the continent and in Greenland, the great majority north of 55°, and migrate in late summer and autumn. The October 1 location of the “falcon front” (the point of peak passage; Worcester & Ydenberg, 2008) is shown (white line). Peregrine passage is much earlier on the Pacific coast, and its timing is much more variable among years than along the Atlantic coast. Western sandpipers (yellow) migrate from breeding areas in coastal Arctic Alaska, beginning with a jump over the Gulf of Alaska to the Pacific Northwest, thereby evading “marine” peregrines of the sub‐species *pealei*. They progress southward to non‐breeding areas along coastlines of the Gulf of Mexico, and Central and South America. This portion of their migration overlaps temporally and spatially with that of southbound peregrines. Their exposure to migrating versus post‐migration resident peregrines varies greatly among years. Semipalmated sandpipers (brown) breed across the Arctic from Alaska to Quebec (dark shade), migrating southeast to major Atlantic coastal staging sites (Bay of Fundy, and Delmarva peninsula, with adjacent Delaware and Chesapeake Bays) where they prepare for the long crossing to non‐breeding areas in South America. Their southward passage precedes that of northern‐breeding migrant peregrines, but they are exposed during several weeks of staging to peregrines introduced during the 1980s and now resident and breeding at these sites.

Western sandpipers (*Calidris mauri*) have also experienced a large increase in the presence of peregrines along their migration route (Ydenberg et al., 2017; Figure 1), though with a significant difference. On the Pacific flyway, peregrines and western sandpipers are co‐migrants on both the northward and southbound migrations (Table 2; Ydenberg, 2022), with the temporal overlap especially large for southbound juveniles. On the Atlantic flyway, in contrast, semipalmated sandpipers have almost completed passage by the time migrant peregrines begin to arrive, and there is thus little southbound co‐migration (Lank et al., 2003, Table 2). However, there are now populations of peregrine pairs breeding at each of their major southward migratory staging areas, populations established by the introduction of captive‐bred peregrines in the 1980s (Dekker et al., 2011; Watts et al., 2015). These peregrines begin breeding before the northbound passage of sandpipers (Watts & Truitt, 2021), and their young have fledged and are hunting by the time of sandpiper southward passage (Dekker et al., 2011).

**TABLE 2:** Attributes of the southbound migration phenology of western and semipalmated sandpipers and peregrine falcons.

Attribute	Western sandpipers	Semipalmated sandpipers
	Pacific flyway	Atlantic flyway
Peregrines primarily encountered^a^ as	Migrants	Breeding residents
Migratory peregrine arrival date (day‐of‐year)^b^	220	273
Inter‐annual range (day)^c^	54	7
Adult sandpiper passage date (day‐of‐year)^d^	198	225
Juvenile passage date (day‐of‐year)^d^	228	250
Headstart^e^	−16–34	48–55
CBC falcon increase 1975–2010^f^	6.5 fold	3.5 fold
Sighting rate (per field h)^g^	~1	~1.8

*Note*: Pacific flyway measurements were made in the Salish Sea of southwest British Columbia (49° N), and Atlantic flyway measurements in the Bay of Fundy (~46° N), except for the CBC falcon increases, which are flyway wide. Dates are reported as “day‐of‐year” (e.g., d‐o‐y 220 = August 8).

^a^ Described in Ydenberg et al. (2022). The peregrines introduced and now breeding along the Atlantic flyway increased from 0 (1975) to 27 pairs (2010) in the Bay of Fundy (Dekker et al., 2011) and even more in Chesapeake Bay (Watts et al., 2015).

^b^ “Arrival” defined as the d‐o‐y on which 50% of the total passage was reached. The inter‐annual average date is given. Based on Worcester and Ydenberg (2008).

^c^ Inter‐annual range (in days) of peregrine arrival dates, based on Niehaus and Ydenberg (2006) for the Salish Sea, and the Bay of Fundy on an analysis of data from nearby North American Hawkwatch sites by Hong Ho (unpubl. report).

^d^ Median passage date varies little between years. Based on Lank et al. (2003), and Niehaus and Ydenberg (2006).

^e^ “Headstart” is the difference (d) between the median peregrine and adult sandpiper passage dates (negative = falcon arrival earlier). Minimum and maximum are given.

^f^ Increase in the total number of falcons counted on North American shorebird wintering sites in Christmas Bird Counts 1975–2010, reported by Ydenberg et al. (2017).

^g^ Peregrine sighting rate per field party hour. Based on Lank et al. (2003) for the Salish Sea; and on Dekker et al. (2011) for the Bay of Fundy.

**FIGURE 2:**
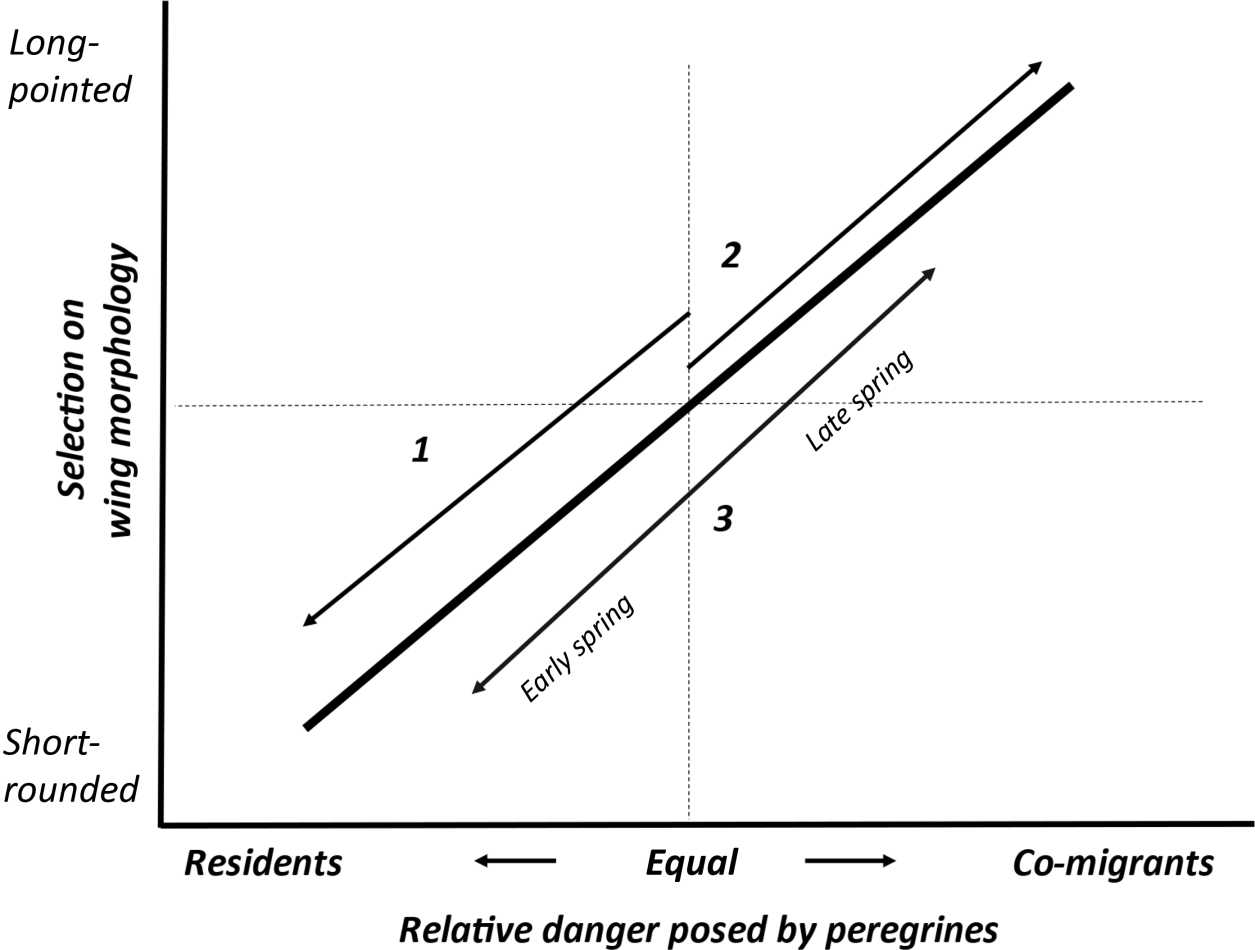
We hypothesize that the selection on wing morphology for long‐distance migrant sandpipers is generated by the relative exposure to resident versus co‐migrating peregrines (horizontal axis). Wing morphology (vertical axis) ranges from short‐rounded (more acceleration and agility) to long‐pointed (more efficient in long‐distance flight). Peregrines are important predators, with strong population recovery ongoing since ~1975. Wing morphology effective at countering this rising danger depends on whether migratory risk is better reduced by escape from attacks at staging sites (#1; semipalmated sandpipers, Atlantic flyway), or by outpacing peregrine migration between stopovers along the route (#2; western sandpipers, Pacific flyway). Relative exposure for western sandpipers also varies between years in direct relation to the variable onset of spring. Accordingly, juvenile wings (grown just prior to migration) are shorter in early spring years, and longer in late spring years (#3). This phenotypic plasticity, superimposed on the long‐term lengthening trend, is interpreted as an induced defense.

Migrant sandpipers require different defenses against co‐migrating versus resident peregrines (Figure 2; Ydenberg et al., 2022). Temporal overlap with migrating peregrines can be reduced by higher migratory speed (Hope et al., 2011, 2014; Ydenberg, 2022; Ydenberg et al., 2007), and also by earlier migratory departure (Jamieson et al., 2014). Sandpipers have up to twice the migratory speed of peregrines (~400 vs. 200 km day^−1^; Hope et al., 2011), so are able to reduce their exposure by advancing more quickly into lower danger zones ahead of migrant peregrines (Ydenberg & Hope, 2019). However, faster or earlier passage does not help to evade resident peregrines. Instead, extra caution is necessary while at staging sites. Over recent decades, migrating semipalmated sandpipers have increasingly aggregated at safer locations (1974–2018; Hope et al., 2020), altered space use (Sprague et al., 2008), and flocking behavior (e.g., Beauchamp, 2008, 2016; Beauchamp & Ruxton, 2008), and have taken up cliff roosting (MacKinnon et al., 2008) and over‐ocean flocking (Dekker et al., 2011) in place of roosting on mudflats at high tide. Lank et al. (2017) suggested that wing shortening be added to this list of anti‐predator adaptations. Here we hypothesize that increasing peregrine populations select either for better escape performance (shorter, rounder, convex wings) or increased migratory speed (longer, pointed, concave wings), depending on the balance of danger posed by exposure to resident versus migratory falcons. Increasing peregrine numbers have made greater migratory speed advantageous on the Pacific flyway, and the hypothesis thus predicts that western sandpipers should have evolved longer rather than shorter wings (Figure 2).

An experiment manipulating the exposure of migrant sandpipers would be impossible on any temporal or spatial scale large enough to be meaningful. However, extensive inter‐annual variation in peregrine migration timing on the Pacific flyway provides a large‐scale natural experiment. The median southward passage timing of peregrines in southern British Columbia varies annually by 54 days (Table 2), shifting with spring onset. Earlier spring increases the proportion of peregrine southward passage preceding that of western sandpipers. Later‐passaging western sandpipers, particularly juveniles, increasingly encounter these peregrines as post‐migration residents settled for the winter (Ydenberg, 2022). The earliness of spring thus shifts the balance of benefits from defense against co‐migrating (longer wings) toward resident peregrines (shorter wings).

Behavioral aspects of defense (e.g., vigilance, stopover site choice, rate, and extent of fueling) are quickly adjusted. Some morphological aspects of defense can also be readily modified (e.g., muscle mass; van den Hout et al., 2010), albeit not as rapidly as behavior. But wing morphology can be adjusted only once annually when flight feathers are grown. Adult western sandpipers do not molt until after southward migration is completed, but juveniles grow their wing feathers before their initial southward migration and could, in principle, tailor wing morphology in anticipation of the type of migratory danger. Changes to defensive morphology induced by (cues to) predator presence are known in many systems (“induced defenses”; Tollrian & Harvell, 1999). We emphasize that doing so does not require juveniles to experience predators directly: they have only to adjust wing growth contingent on a cue relevant to migratory predation danger.

The timing of spring onset provides such a cue (Ydenberg, 2022). Spring onset is a marked and rapid event on the Arctic breeding grounds of shorebirds, widely measured by snowmelt (e.g., van Gils et al., 2016). Juveniles do not experience this directly as they are (necessarily) hatched after snowmelt, but there are many possible ways for them to assess this (e.g., by vegetation development in relation to date at hatch). Mothers could even transmit a signal (“trans‐generational plasticity”, or TGP; MacLeod et al., 2022) to the developing embryo. In a paper entitled “… a pathway to translate predation risk to offspring”, Coslovsky et al. (2012) found that experimentally exposing great tit mothers to predators during laying altered levels of maternal steroids in the egg yolk, which in turn affected the growth and behavior of offspring—including the growth of longer wings.

We predict, therefore, that superimposed on the lengthening trend of wings, there is an additive component adjusting the annual mean wing length of juvenile (but not adult) western sandpipers, in direct relation to the date of spring onset. This mechanism favors shorter wings when spring is earlier due to the greater relative exposure to resident peregrines, and longer wings following later springs when relatively more exposure to co‐migrating peregrines is expected.

## 2 METHODS

We measured wing (flattened wing chord of the longest primary; to 1.0 mm) and culmen (bill; to 0.1 mm) lengths of southbound migrant western sandpipers captured on stopover in southwestern British Columbia, 1978–2020 (*n* = 12,134; 4719 adults in 23 years; 7415 juveniles in 22 years). We also measured body mass (to 0.1 g), but this is a poor measure of size for migrant birds due to large and rapid changes in fat load during stopover. Substantial sample sizes were obtained between 1978 and 2001 in all but 4 years for adults and 2 years for juveniles (Appendix A) as part of a Canadian Wildlife Service program. Post‐2001 capture effort was opportunistic, irregular, and much smaller.

Many different observers made measurements, and we treat this source of error in Appendix B. The complications of interpreting morphological change at stopover sites that arise when populations with different body sizes mix (as in semipalmated sandpipers; Lank et al., 2017) are not an issue, because western sandpipers have a compact breeding range with little or no morphological or genetic structuring (Schwarz, 2016).

To assess the relationship between wing length and shape, we quantified shape as the second size‐constrained component (C2; Swaddle & Lockwood, 1998), calculated from measurements of all 10 primaries of western sandpipers measured in Mexico, January–March of 1999 and 2000 (*n* = 506, see Fernández & Lank, 2007). To plot comparisons on common axes, we calculated size‐constrained components for samples pooled across age and gender. Birds were gender‐assigned based on culmen length (males and females differ substantially). For comparison, we present a previously‐unpublished parallel analysis of semipalmated sandpiper wing length and shape, based on measurements made in Perú in January–March 2015 (*n* = 125).

We modeled temporal trends in wing and bill length as linear functions of the year. There were no interactions with gender, and we thus calculated annual means of gender residuals, added global age‐specific means to rescale, and estimated long‐term trends as linear regressions on annual means, weighted by 1/annual residual variance to account for substantial differences in annual sample sizes. Because limited data are available after 2001, we also present results from simple linear regression modeling rates of change in age‐specific annual means for 1978–2001 only. For consistency with our previous analysis of wing length change in migratory semipalmated sandpipers (Lank et al., 2017), we restricted this latter analysis to years in which western sandpiper sample sizes in an age class exceeded 20 and were gathered over at least a 19 day span. To compare overall trends between western and semipalmated sandpipers, we simplified the analysis of the species presented by Lank et al. (2017) covering 1980–2015, which considered eastern, central, and western breeding regions (based on Gratto‐Trevor et al., 2012) trends separately. Since there were no regional differences in slopes, we here produce a single trend line by adjusting the mean values for regional size differences with an ANOVA and modeling the adjusted wing lengths as a linear function of year.

We assessed the relationship of annual mean western sandpiper wing length to spring onset timing, based on snowmelt in western sandpiper breeding areas, following the method described by van Gils et al. (2016). We estimated snowmelt for 1978–2001 using their function and fitting procedure (described in their Supplementary Materials: Methods, and with code accessed from their data Dryad https://doi.org/10.5061/dryad.n1m8d). We used snowmelt data from the NOAA CDR climate dataset on a resolution of 33 × 35 km (downloaded from https://www.ncei.noaa.gov/thredds/catalog/cdr/snowcover/catalog.html), clipped and resampled to the western sandpiper breeding range map from BirdLife International (2018). We re‐projected the snow cover and breeding range maps as stereographic projections centered around 64° N, 163° W to avoid shape distortion. The snowmelt date was determined as the date on which the fitted curve predicted that 25% of the western sandpiper breeding range would be snow‐free. This threshold is slightly lower than van Gils et al. (2016) because western sandpipers breed mainly near the coast and are less influenced by later interior snowmelt. We restricted the analysis to years in which western sandpiper sample sizes in an age class exceeded 20 and were gathered over at least a 19 day span (see Lank et al., 2017). Annual gender‐adjusted wing and bill length means were modeled for both age classes as linear functions of year and the timing of snowmelt for 1978–2001.

## 3 RESULTS

**FIGURE 3:**
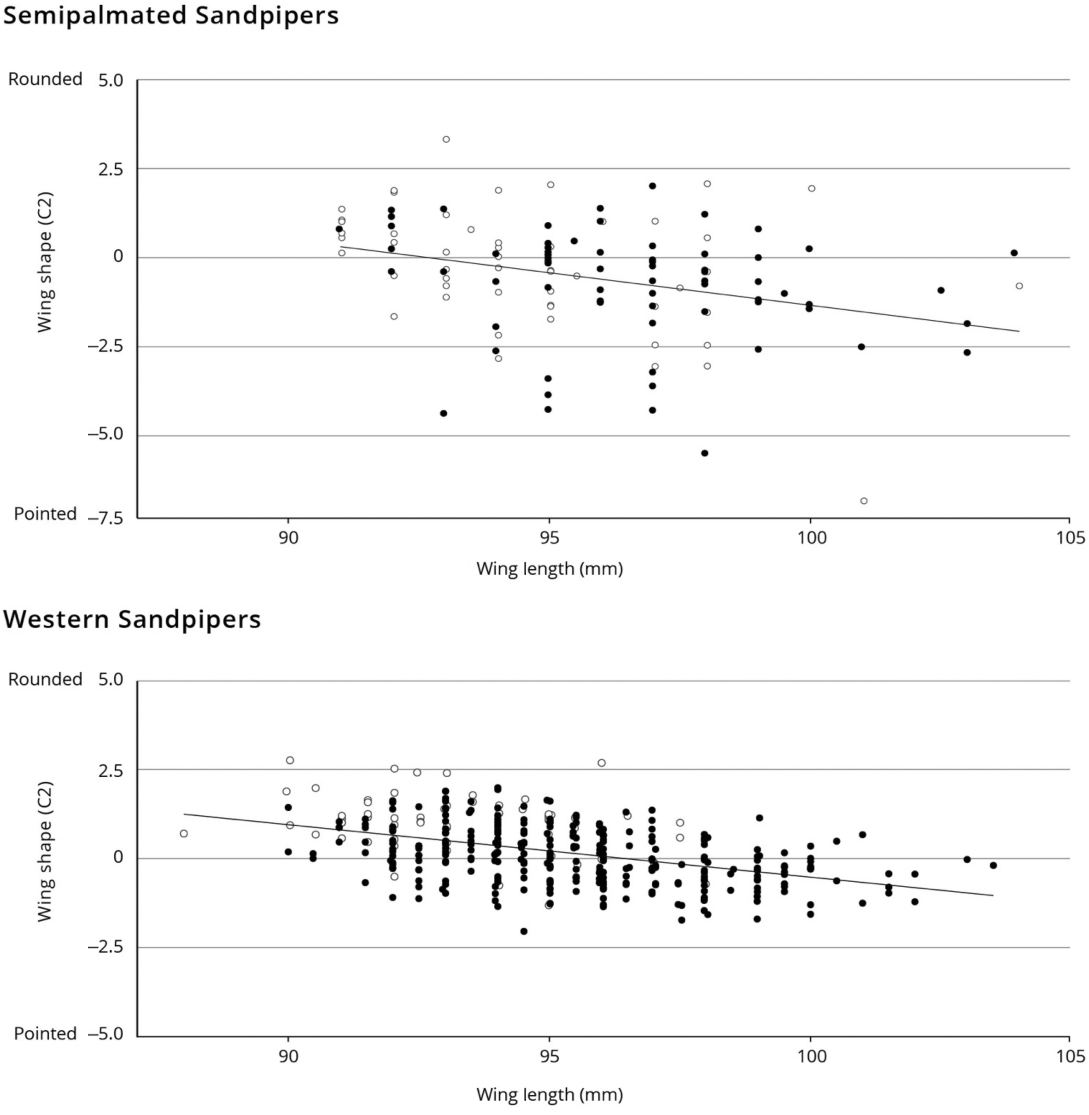
Relationship between wing shape (principal component C2) and wing length (flattened wing chord) in semipalmated (upper panel) and western sandpipers (lower). The axis of intraspecific variation in both species lies from short, rounded wings (upper left) to long, pointed wings (lower right), for adults (solid dots) and juveniles (open dots). Slopes are given in principal component C2 units per mm of wing length. There are no significant differences in slope between age or sex classes within either species. The semipalmated sandpiper relationship is based on unpublished feather length measurements taken in Perú in January–March 2015, *N* = 125 wings. Both species are portrayed here to illustrate their similarity. Semipalmated sandpiper: slope = −0.18 mm^−1^, *r* ^2^ = .11, *F* = 13.90, *p* < .0003. Western sandpiper: slope = −0.15 mm^−1^, *r* ^2^ = .20, *F* = 93.84, *p* < .0001.

Intraspecific morphological variation of western and semipalmated sandpiper wings is similar and lies along an axis ranging from relatively short‐and‐rounded to relatively long‐and‐pointed wings (Figure 3). The flattened, straightened wing length, therefore, provides a reasonable measure representing wing size and shape for both species.

**FIGURE 4:**
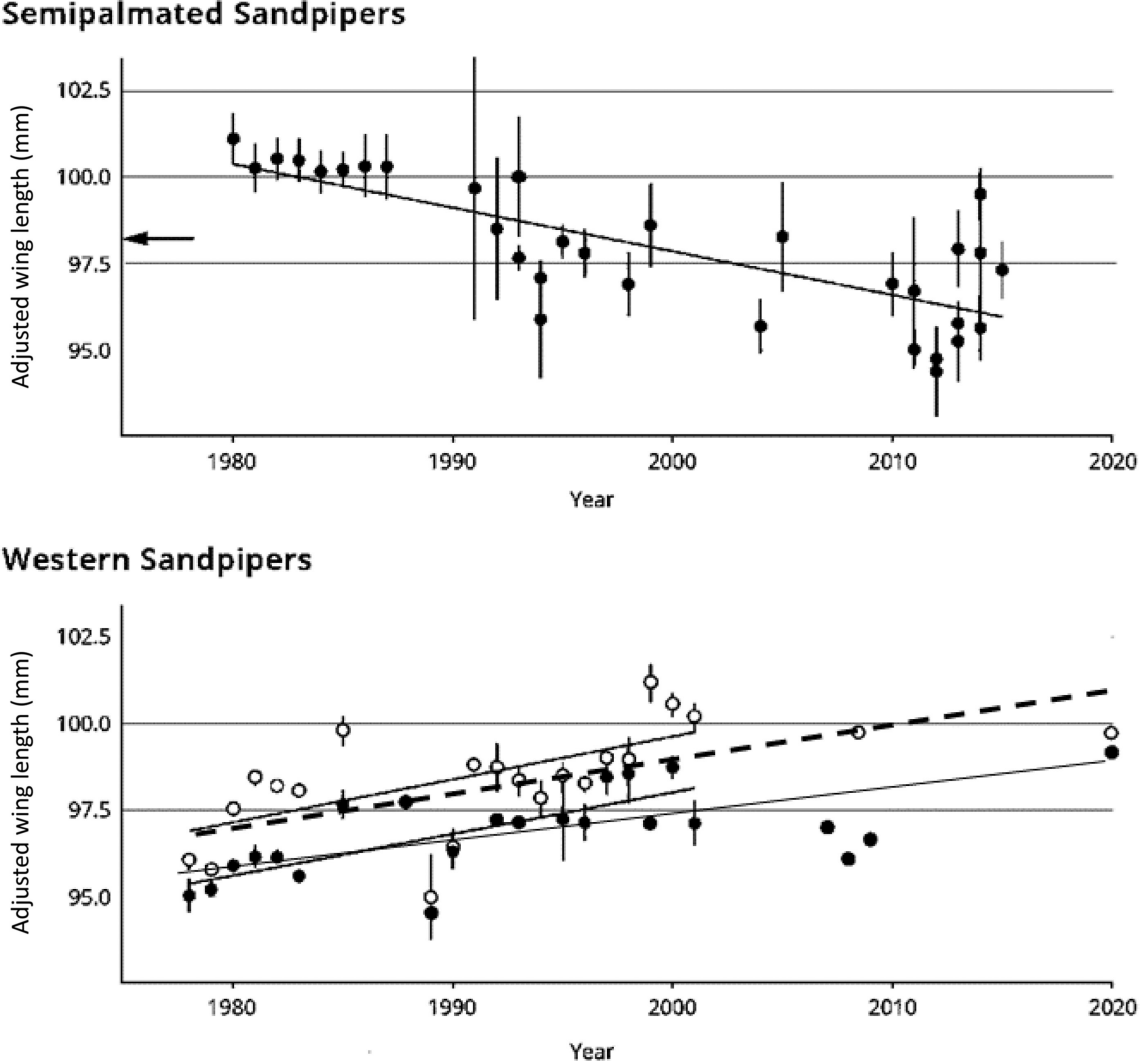
Wing length (mm; flattened wing chord) change in long‐distance migrant sandpiper species over recent decades. (A) Semipalmated sandpipers (*n* = 1492 adults measured at five breeding sites 1980–2015, from Lank et al., 2017). Depicted are 32 site‐year mean values, adjusted for size differences among breeding regions, based on Coslovsky et al. (2012), see Section 2. Wing length decreased steadily at an overall rate of −0.103 mm year^−1^ (SE = 0.016, *F* = 40.09, 1 *df*, *p* < .0001), based on a simple regression on mean values. Bars are standard errors of annual values. The arrow indicates the mean value of shrinkage‐adjusted museum specimens collected prior to the 1970s (Lank et al., 2017). (B) Western sandpipers (*n* = 12,134 individuals measured on southward migration in southwest British Columbia, 1978–2020). Depicted are 23 adult and 22 juvenile gender‐adjusted annual means; bars are 95% CIs. Annual mean wing length increased significantly over all years, with the adult rate estimated at 0.074 mm year^−1^ (SE = 0.017; *p* < .0003, closed black dots with solid line), and the juvenile rate at 0.087 mm year^−1^ (SE = 0.029; *p* < .007, open black dots with dashed line). Simple regressions on mean values for 1978–2001 are also shown, excluding points with small sample sizes (see Appendix A).

Wing length (annual means, controlling for gender) of western sandpipers increased significantly from 1978 to 2020 at 0.074 mm year^−1^ (SE = 0.017; *p* < .0003) for adults (23 years); and 0.087 mm year^−1^ (SE = 0.029; *p* < .007) for juveniles (22 years) (Table 3, Figure 4). If restricted to years between 1978 and 2001 with large samples and adequate date range (Appendix A), the rates are 0.11 mm year^−1^ for adults (*n* = 4485 wings, measured in 15 years), and 0.12 mm year^−1^ for juveniles (*n* = 7263 wings, 18 years). In contrast, adult semipalmated sandpiper wing length decreased (1980–2015) at an overall rate estimated at −0.103 mm year^−1^ (SE = 0.016, *p* < .0001, Figure 4). Over the decades, these produced 4–5% changes. In contrast, the rates of change in annual mean bill lengths of both species are statistically indistinguishable from zero (Table 3). Thus in neither species can the change in wing length be explained as part of a general response correlated with overall body size change.

**TABLE 3:** The rate of change in mean annual wing and bill lengths (mm year^−1^) of adult (*n* = 23 years) and juvenile (*n* = 22 years) southbound migrant western sandpipers captured and measured on the Fraser estuary, British Columbia, between 1978 and 2020.

	Rate	SE	*t*	*p*
Adult wings	0.074	0.017	4.31	.001
Juvenile wings	0.087	0.029	3.02	.007
Adult bills	0.002	0.003	−0.59	.560
Juvenile bills	0.002	0.004	0.45	.066

*Note*: Slopes were calculated on means weighted by 1/residual annual variance, to account for large differences in annual sample sizes (Appendix A). Wings of both age classes lengthen significantly at similar rates, while bills do not change.

**FIGURE 5:**
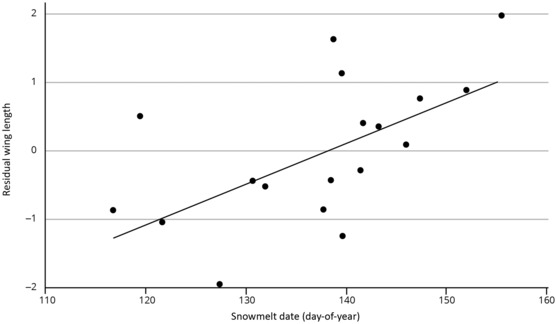
The wing length of southbound juvenile western sandpipers shortens in relation to the snowmelt date on the breeding area in Alaska (1978–2001). The points are residuals of expected annual means from a model with year as a linear term and controlled for gender. The effect of snowmelt date is thus additive to the lengthening effect of the year, with wing length shortening significantly by 0.055 mm for each day that snowmelt is earlier (*r* ^2^ = .38, *p* = .008).

The relationship between snowmelt date and juvenile western sandpiper annual residual mean wing length is portrayed in Figure 5. Controlling for the long‐term trend, snowmelt date shows the predicted effect, with residual juvenile wing length negative in years with early spring onset and positive when later. A model predicting juvenile wing length (annual mean gender‐controlled residual) as a linear function of year and snowmelt date estimates that wings are shorter by 0.055 mm for each day that snowmelt is earlier (*r* ^2^ = .38, *p* = .008). No such effect is detectable for adults (*r* ^2^ = .08, *p* = .32). The analogous model for culmen length shows no effect for either age class (*p*‐values > .20).

## 4 DISCUSSION

The data presented here show that the wings of western sandpipers lengthened steadily and substantially between 1978 and 2020, coincident with the recovery of peregrine falcon population numbers following the mid‐1970s regulation of DDT. Wing length of the ecologically‐similar semipalmated sandpiper shortened by about the same amount during this period (1980–2015). Based on measurements of 137 museum specimens collected prior to the mid‐1970s, Lank et al. (2017) also showed that semipalmated sandpiper wings lengthened after DDT came into widespread usage, reaching a maximum in 1980, before beginning to shorten (Figure 4). They argued that as peregrine populations were decimated, escape performance declined in importance relative to flight performance. The effect of changes of this magnitude on flight performance is substantial: based on Program Flight (Version 1.14; Pennycuick, 1989), an increase in wing length of 4 mm adds ~25% to the flight range of a 25 g male western sandpiper.

Note that the absolute rates of change measured in western and semipalmated sandpipers (0.074–0.110 mm year^−1^) are at least an order of magnitude larger than those reported in other studies of wing length change (see Table 1), which range from ~0.001 to 0.005 mm year^−1^. However, when expressed as “allochronic” phenotypic rates of change (estimated at 990 and 1100 darwins, for western and semipalmated sandpipers, respectively), they fall within the range of other studies reporting the impact of predators on morphology (Hendry & Kinnison, 1998).

In addition to documenting decade‐scale changes in wing lengths, we tested the hypothesis that wing length changes are responses to peregrine exposure with an analysis of annual wing size variation of juvenile western sandpipers, predicted based on the idea of an induced defense. Large climate variability (especially in spring onset timing) is a natural feature of the northeastern Pacific and strongly affects the phenology of endemic fauna (e.g., timing of breeding in Cassin's Auklet *Ptychoramphus aleuticus*; Bertram et al., 2001). There is extensive inter‐annual variability in the relative timing of western sandpiper and peregrine breeding cycles and thus in the degree of temporal migratory overlap (Ydenberg, 2022). This creates an annually‐repeated large‐scale natural experiment (Barley & Meeuwig, 2017) with which to challenge the “co‐migrating vs. resident predator” hypothesis. In years with an early spring, peregrine migration is well underway as western sandpipers depart breeding areas. This shifts their relative exposure away from co‐migrants toward post‐migratory peregrines that take up winter residency along the Pacific flyway (Ydenberg, 2022).

Juvenile western sandpipers grow wings just before their southward migration, and as predicted (Figure 1), the residual (relative to the lengthening inter‐annual trend) annual mean wing length changes in direct relation to snowmelt date (Figure 5). Our measures were made at the first major migratory stopover, and there is little exposure to peregrines or other predators along the route prior to this point (Ydenberg et al., 2022). The changes in residual wing lengths can therefore not be attributed to direct killing and must have arisen prior to embarking on migration.

The ecology of juvenile western sandpipers shows pertinent features parallel to that of crucian carp *Carassius carassius*, a model system for the study of induced defenses (Tollrian & Harvell, 1999). Exposure during early life to the odor of pike (*Essox lucius*; Domenici et al., 2008) induces a deeper body form that reduces vulnerability (pike are gape‐limited) and improves escape performance (higher speed, acceleration, and agility). Such phenotypic plasticity is adaptive because the abundance of pike varies greatly and unpredictably between years, and is signaled by cues available before specific morphology is adopted. Because the defensive body form reduces cruising ability and hence foraging performance, the regular phenotype is preferable in the absence of pike. Juvenile western sandpiper ecology shares these features: the relative danger posed by resident versus co‐migrating peregrines along the Pacific flyway varies substantially and irregularly among years; the timing of snowmelt on breeding grounds provides a reliable cue that occurs before flight feathers are grown; and longer wings are preferable in years with later snowmelt because they help boost migratory speed.

Recent interest in climate change has stimulated an extensive literature interpreting patterns of morphological change as thermoregulatory responses to higher ambient temperature (e.g., Gardner et al., 2011, 2014; see also Baudron et al., 2014; Rode et al., 2010; Weeks et al., 2019). Wings generally have no thermoregulatory function in birds (Swaddle & Lockwood, 1998), so studies invoking climate change as the cause (studies 1–5 in Table 1) usually interpret changes in their length as responses correlated with general body size change. There is also much interest in morphological change resulting from climate‐change‐induced food shortages (Sheridan & Bickford, 2011). van Gils et al. (2016) present an example, showing that the body (bill) size of juvenile knots (a shorebird; *Calidris canutus*) decreased over a 25‐y period as the synchrony of their Arctic breeding season with peak food availability diminished due to climate change (“ecological mismatch”).

General body size change could result from size selection by predators, or be due to its effects on availability, vulnerability, or escape performance (Andraso & Barron, 1995; Domenici et al., 2008; McCollum & Leimberger, 1997; O'Steen et al., 2002; Reimchen, 1994; Reimchen & Nosil, 2004; Reznick et al., 1990). General body size change could also arise as a by‐product of behavioral or physiological changes made to increase (or lower) safety in ways that also affect growth rates, such as more (less) cautious foraging (e.g., DeWitt et al., 2019) or a habitat shift (e.g., Swain et al., 2015). However, we could detect no change in another structural measure of body size (bill length) in either species, as would be expected if the change in wing length were attributable to general body size change. We also note that the interval between the hatch date of juvenile western sandpipers and their southward passage on the Fraser estuary (on average 54.3 ± 3.6 days) lengthens when the hatch is earlier (Niehaus & Ydenberg, 2006), suggesting that the growth period is if anything longer in early snowmelt years. Nonetheless wing lengths are shorter, opposite to what would be expected if the inter‐annual variability resulted from a temporal constraint on primary feather growth prior to migratory departure.

In conclusion, we attribute the extensive and rapid changes in opposing directions of sandpiper species' wing lengths recorded since ~1980 to heightened migratory danger posed by the ongoing recovery of peregrine falcons. The changes seem likely to be due to selection on wing size and shape, both of which strongly affect flight performance attributes. That phenotypic plasticity also plays a role is suggested by the annual change in the (residual) wing length of juvenile western sandpipers, consistent with defensive morphology induced by cues to the relative exposure during migration to resident versus co‐migrating peregrines. The fundamental trade‐off in wing morphology pits energetic efficiency (enhanced by longer, pointier, concave wings) against flight performance attributes such as acceleration and agility (enhanced by shorter, rounder, convex wings). The rate and direction of wing length change is due to changes in the relative importance of flight performance attributes as predator abundance climbed. Raptor species other than peregrines have also shown substantial increases in abundance since the mid‐1970s, a trend that contrasts strongly with the decline shown by most other groups of birds in North America (Rosenberg et al., 2019). For many prey species, therefore, the past half‐century spans a period of steadily increasing danger, suggesting that the hypothesis developed here could apply more broadly.

## AUTHOR CONTRIBUTIONS

**Ronald C. Ydenberg:** Conceptualization (lead); data curation (supporting); formal analysis (equal); funding acquisition (lead); methodology (equal); writing – original draft (lead); writing – review and editing (equal). **Guillermo Fernández:** Data curation (supporting); formal analysis (supporting). **Enver Ortiz Lopez:** Data curation (supporting). **David B. Lank:** Conceptualization (equal); data curation (lead); formal analysis (equal); methodology (equal); software (equal); writing – original draft (equal); writing – review and editing (equal).

## Data Availability

The DOI for Dryad data archive: https://doi.org/10.5061/dryad.kd51c5b51.

## APPENDIX A

This Appendix summarizes measures of adult semipalmated and adult and juvenile western sandpipers. Semipalmated sandpipers listed by breeding region (eastern, central, or western Arctic) in which adults were trapped. Western sandpipers were all captured on migration in southwestern British Columbia. The “region‐adjusted” wing length of semipalmated sandpipers corrects for differences in body size (Coslovsky et al., 2012) among the breeding regions. The “sex‐adjusted” measures of western sandpipers correct the difference in male and female body size.

**Table:**

Adult breeding Semipalmated sandpipers						
Year	Region	*n* birds	× Wing (mm)	× Region‐adjusted wing (mm)	Lower 95% CI	Upper 95% CI
**<1970**	**All**		**97.8**			
1980	Eastern	40	101.10	99.97	100.38	101.82
1981	Eastern	52	100.25	99.12	99.56	100.94
1982	Eastern	60	100.52	99.39	99.90	101.13
1983	Eastern	55	100.47	99.34	99.84	101.11
1984	Eastern	54	100.15	99.02	99.54	100.75
1985	Eastern	66	100.20	99.07	99.67	100.73
1986	Eastern	33	100.30	99.17	99.41	101.20
1987	Eastern	28	100.29	99.16	99.37	101.20
2004	Eastern	35	95.69	94.56	93.77	95.35
2005	Eastern	15	98.27	97.14	95.55	98.72
2013	Eastern	34	97.91	96.78	95.68	97.89
2014	Eastern	28	99.50	98.37	97.63	99.11
2015	Eastern	48	97.31	96.18	95.37	97.00
1991	Central	3	99.67	99.67	95.87	103.46
1992	Central	8	98.50	98.50	96.45	100.55
1994	Central	11	100.00	100.00	98.27	101.73
2010	Central	9	95.89	95.89	94.19	97.58
2011	Central	14	96.70	96.70	94.60	98.83
2012	Central	16	94.38	94.38	93.07	95.68
2013	Central	28	95.25	95.25	94.10	96.40
2014	Central	27	97.80	97.80	95.49	100.14
1993	Western	194	97.66	98.20	97.29	98.02
1994	Western	131	97.08	97.62	96.64	97.51
1995	Western	118	98.13	98.67	97.65	98.61
1996	Western	46	97.80	98.34	97.11	98.49
1998	Western	35	96.90	97.44	95.98	97.82
1999	Western	20	98.60	99.14	97.39	99.81
2010	Western	32	96.91	97.45	96.53	98.36
2011	Western	90	95.02	95.56	94.99	96.12
2012	Western	69	94.74	95.28	94.56	96.00
2013	Western	58	95.77	96.31	95.65	96.96
2014	Western	32	95.64	96.18	95.23	97.13

Migrant Western Sandpipers												
Year	Juveniles (open circles)						Adults (closed circles)					
	*n* birds	× Sex‐adjusted wing (mm)	Lower 95% CI	Upper 95% CI	× Bill (mm)	× Sex residual bill	*n* birds	× Sex‐adjusted wing (mm)	Lower 95% CI	Upper 95% CI	× Bill (mm)	× Sex residual bill
1978	407	96.206	95.969	96.443	24.701	−0.228	124	94.785	94.291	95.279	24.206	−0.018
1979	726	95.926	95.755	96.097	24.524	−0.158	498	95.014	94.776	95.252	25.258	0.163
1980	783	97.674	97.486	97.861	24.291	−0.214	1131	95.645	95.459	95.831	24.005	−0.049
1981	579	98.580	98.374	98.785	24.378	−0.054	307	95.872	95.541	96.203	23.519	−0.028
1982	1754	98.337	98.231	98.444	24.701	0.132	778	95.889	95.687	96.091	23.894	−0.111
1983	1335	98.192	98.046	98.337	24.537	0.106	703	95.332	95.127	95.536	23.811	−0.054
1985	90	99.937	99.498	100.380	24.261	−0.154	220	97.405	96.998	97.812	24.233	0.056
1988	21	97.529	96.704	98.355								
1989	57	94.329	93.680	94.977			43	94.820	93.601	96.040	25.895	0.368
1990	133	96.579	96.025	97.132	25.387	0.544	106	96.057	95.541	96.573	24.559	0.344
1992	39	98.910	98.202	99.618	25.141	0.120	14	98.425	96.760	100.090		
1993	142	98.483	98.067	98.899	24.740	0.146	19	97.345	95.958	98.732		
1994	93	97.967	97.424	98.509	24.051	−0.081	8	97.194	95.496	98.892		
1995	142	98.621	98.190	99.052	24.250	−0.102	35	96.957	95.764	98.150	23.691	0.035
1996	411	98.412	98.183	98.642	24.618	−0.076	97	96.849	96.321	97.378	23.270	−0.143
1997	243	99.126	98.838	99.413	24.846	0.255	126	98.227	97.709	98.745	24.583	0.017
1998	100	99.064	98.381	99.747	23.961	−0.039	38	98.292	97.451	99.134	24.207	0.152
1999	59	101.310	100.760	101.860	24.642	0.246	23	97.232	96.189	98.274		
2000	156	100.690	100.340	101.040	24.617	−0.034	205	98.468	98.129	98.808	23.864	−0.081
2001	71	100.320	99.892	100.760	24.704	−0.010	74	96.868	96.210	97.527	24.280	0.103
2007							60	97.176	96.532	97.819		
2008	14	99.524	98.162	100.890			30	96.440	95.518	97.361		
2009							60	96.802	96.251	97.352		
2020	60	99.526	98.974	100.080			20	99.056	98.036	100.080		
**All**	**7415**						**4719**					

*Note*: The sole bolded value in the upper (semipalmated sandpiper) portion of the table gives the mean wing length of pre‐1970 museum specimens. This sample is a mixed bag, with the collection date of many unclear, other than they must predate 1970. No significance can properly be assigned to this estimate. The bolded values in the lower portion are simple total sample sizes.

## APPENDIX B

### WING LENGTH MEASUREMENT ERROR

During the review process, several referees commented that the measurement error is large, and raised the possibility that the changes we report are spurious. In this Appendix, we enlarge on the analysis of measurement error included in our previous paper on semipalmated sandpiper wing length change (Lank et al., 2017, p. 3251) for which a similar concern was raised.

Wings were measured to the nearest 1.0 mm (0.5 mm at some sites), and multiple observers contributed measures to both the semipalmated (Lank et al., 2017) and western sandpiper (this paper) studies. Measurement error for semipalmated sandpiper wings was assessed using data on 17,035 birds captured at James Bay, with measures made by 10 different observers 1975–1982 (368–2453 birds/observer/year). The mean deviation of each observer from the global mean was calculated: seven of the 10 observers fall within 0.5 mm of the global mean. The mean difference between the 45 possible pairs of observers is 0.53 mm (maximum difference 1.6 mm). Independent measures of the same bird within a single season (*n* = 35) were used to estimate the intraclass correlation coefficient (i.e., repeatability, cf. Lessells & Boag, 1987). That value is 0.85. Lank et al. (2017) also report that substantial change was measured within sites despite having consistent investigator training, and even in one case when all birds were measured by a single observer.

Measurement error in western sandpipers was assessed by having five experienced observers measure each of 16 western sandpipers in a blinded procedure conducted at Roberts Bank, British Columbia in August, 2014. The mean difference between the 10 possible pairs of observers was 0.97 ± 0.65 (*SD*) mm. Repeatability was 0.62, with an observer variance component of 0.52. Thus 67% of observations between observers are expected to fall within 0.72 mm (±1 *SD*) of the mean, and 95% of values within 1.44 mm (±2 *SD*).

These assessments show though individual observers have consistent differences, their measures tend to lie close (within 1 mm) together, which is about equal to the accuracy of measurement. We conclude that measurement error is substantially less than the effect size. Moreover, most of the annual means reported in the papers are composed of measurements made by multiple individuals, whose individual biases would tend to offset rather than reinforce one another. Both studies show highly significant linear trends spanning 36–42 years, with data collected in many (>20 in each case) of the years, and overall change estimated at 3.5–4 mm, making it highly unlikely that the effect can be attributed to measurement biases or errors.

Finally, we point out again that the rates of wing length change measured in these two species (0.074–0.110 mm year^−1^) are at least an order of magnitude larger than those reported for other species in Table 1 (~0.001–0.005 mm year^−1^). As the basic measurement procedure and instruments are the same, the size of these rates can be expected to have made them easier to detect, and less likely to be attributable to measurement error.
