# Correction to Avian wings can lengthen rather than shorten in response to increased migratory predation danger

**DOI:** 10.1002/ece3.10432

**Published:** 2023-08-29

Ronald C. Ydenberg, Guillermo Fernández, Enver Ortiz Lopez, David B. Lank

First published: 22 July 2023.

https://doi.org/10.1002/ece3.10325

In the table description in Appendix A, the reference to Coslovsky et al. (2012) was incorrect. It should read Gratto‐Trever et al. (2012).

We apologize for this error.

